# Biosensors Based on Phenol Oxidases (Laccase, Tyrosinase, and Their Mixture) for Estimating the Total Phenolic Index in Food-Related Samples

**DOI:** 10.3390/life13020291

**Published:** 2023-01-20

**Authors:** Aleksey Tarasov, Natalia Stozhko, Maria Bukharinova, Ekaterina Khamzina

**Affiliations:** 1 Scientific and Innovation Center of Sensor Technologies, Ural State University of Economics, 8 Marta St., 62, 620144 Yekaterinburg, Russia; 2 Department of Physics and Chemistry, Ural State University of Economics, 8 Marta St., 62, 620144 Yekaterinburg, Russia

**Keywords:** total phenolic content, total phenolic index, laccase, tyrosinase, enzymatic biosensor, bienzymatic biosensor, polyphenol oxidase, food analysis, food control, food quality

## Abstract

Plant phenolic compounds demonstrate bioactive properties in vitro and/or in vivo, which creates demand for their precise determination in life sciences and industry. Measuring the concentration of individual phenolic compounds is a complex task, since approximately 9000 plant phenolic substances have been identified so far. The determination of the total phenolic content (TPC) is less laborious and is used for the qualimetric evaluation of complex multicomponent samples in routine analyses. Biosensors based on phenol oxidases (POs) have been proposed as alternative analytical devices for detecting phenolic compounds; however, their effectiveness in the analysis of food and vegetal matrices has not been addressed in detail. This review describes catalytic properties of laccase and tyrosinase and reports on the enzymatic and bienzymatic sensors based on laccase and tyrosinase for estimating the total phenolic index (TPI) in food-related samples (FRSs). The review presents the classification of biosensors, POs immobilization, the functions of nanomaterials, the biosensing catalytic cycle, interference, validation, and some other aspects related to TPI assessment. Nanomaterials are involved in the processes of immobilization, electron transfer, signal formation, and amplification, and they improve the performance of PO-based biosensors. Possible strategies for reducing interference in PO-based biosensors are discussed, namely the removal of ascorbic acid and the use of highly purified enzymes.

## 1. Introduction

### 1.1. Diversity and Significance of Plant Phenolic Compounds

Phenolic compounds as secondary metabolites can be found in all plants. They protect plants from abiotic and biotic stresses caused by unfavorable environmental factors and pathogens. Some phenolic compounds are also involved in coloring different parts of plants and are responsible for organoleptic properties of plant food [1,2,3,4]. According to the structure of the carbon chain, phenolic compounds can be divided into 16 classes [3]. However, the most essential for human diet phenolic compounds are phenolics acids, flavonoids, tannins, stilbenoids, and lignans. Flavonoids are the largest class of phenolic compounds and include major flavonoids (2-phenylbenzopyrans), isoflavonoids (3-benzopyrans), neoflavonoids (4-benzopyrans), and minor flavonoids [4]. Currently, the number of phenolic substances is about 9000 [5], of which more than 6500 compounds fall on the share of flavonoids, and it is assumed that the number of the latter may exceed 8000 [4].

**Figure 1:**
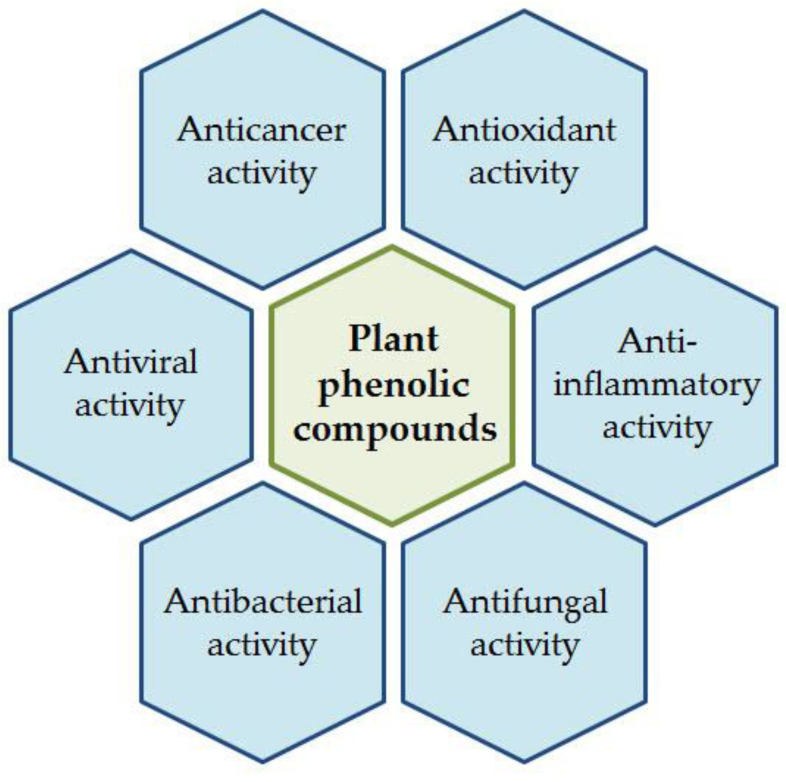
Some types of biological (pharmacological) activities of plant phenolic compounds [2,3,5,6,7,8,9,10].

Phenolic compounds of plant origin have antioxidant, anti-inflammatory, anticancer, antiviral, antibacterial, antifungal, antidiabetic, cardioprotective, hepatoprotective, neuroprotective, and other effects in vitro and/or in vivo [2,3,5,6,7,8,9,10] (Figure 1). This enables us to look at plant phenolic compounds as potential therapeutic agents against pathological conditions and human diseases caused by oxidative stress, pathogens, inflammation, and aging. By rough estimates, there are about 200 diseases associated with a higher level of oxidative stress [11], and over 1400 species of bacteria, fungi, viruses, protozoa, and helminths are known to be able to infect humans [12]. Systemic chronic inflammation is involved in the pathogenesis of cancer, diabetes mellitus, ischemic heart disease, stroke, non-alcoholic fatty liver disease, chronic kidney disease, and autoimmune and neurodegenerative conditions [13]. The consumption of some flavonoids contributes to a prolonged lifespan of model organisms (worms, flies, and even some mouse strains), which makes it possible to consider these organisms as potential candidates for geroprotectors [14]. Dietary phenolic compounds have not yet received the status of essential nutrients, as their health-promotion properties are under study and require strong clinical evidence. Together with carotenoids and other dietary phytochemicals, they are included in the class of phytonutrients [15]. However, there is substantial epidemiological evidence that prudent consumption of polyphenol-rich fruits, vegetables, spices [16], berries [17], tea [18], coffee [19], olive oil [20], and other functional plant-based foods is beneficial for human health.

In recent years, the identification, extraction, purification, determination, and study of bioactivity and increased bioavailability of plant phenolic compounds have spurred much research in life sciences. The bioactivity of phenolic compounds from food plants and herbs allows for their use in food [21,22], pharmaceutical [7,21], and cosmetic [21,23] industries. On an industrial scale, individual phenolic compounds in the form of isolates, as well as phenolic compositions in the form of extracts and premixes, can be used. In the latter case, the qualimetric evaluation of the target ingredient (phenolic composition) and the finished product can be performed by determining the total concentration of phenolic compounds. In particular, phenolic extracts are used in the development of fortified foods [24], functional foods [25], sports nutrition [26], and dietary supplements [27]. These data indicate the importance of monitoring phenolic compounds not only for research but also for production purposes.

### 1.2. Estimation of Total Phenolic Content

There are two analytical approaches to characterize the phenolic status of a sample: (1) measuring the concentration of individual phenolic compounds, and (2) estimating the total phenolic content (TPC). The first approach is a complex task, since about 9000 plant phenolic compounds have been identified so far [5]. The second approach is less laborious and can be recommended for the qualimetric evaluation of complex multicomponent samples in routine analyses. In complex multicomponent matrices, phenolic compounds can interact with each other, as well as with other organic and inorganic substances. When phenolic compounds interact with each other, this may result in the formation of dimers, polymers, and stable intermolecular complexes of phenols [28]. When phenolic compounds interact with other organic and inorganic substances, such as peptides, proteins, lipids, carbohydrates [28,29], and metal cations [30,31], this interaction might be of a covalent or non-covalent nature. Thus, the TPC may present additive value in case of non-interacting phenolic compounds or cumulative (integrated) value in case of interacting phenolic compounds. In food matrices, where the interaction between different components is common, the latter case is more likely. Obviously, these interactions impact the nutritional and functional properties of food products. Analytical methods for evaluatingthe TPC include ultraviolet-visible spectrophotometry [32,33,34], near-infrared spectroscopy (NIRS) [35,36,37], Fourier transform near-infrared spectroscopy (FT-NIRS) [38,39,40], fluorescence spectroscopy [41,42,43], and high-performance liquid chromatography (HPLC) [44,45].

Spectrophotometric analysis with the use of the Folin-Ciocalteu reagent is a well-known method for evaluating the TPC. The Folin-Ciocalteu assay (FCA) is based on the oxidation of polyphenols with a phosphomolybdenum reagent in a carbonate-alkaline aqueous solution, which results in a blue-colored product with a broad absorption maximum between 750 and 765 nm [32]. FCA has been recognized by the Association of Official Agricultural Chemists (AOAC) International as an official method [33]. However, non-phenolic antioxidants and other reducing species present in the analyzed sample are also capable of being oxidized by the Folin-Ciocalteu reagent and lead to unwanted positive interference. In food and vegetal matrices, the potential interfering agents are amino acids, peptides, proteins, reducing sugars, aromatic amines, nitrogenous bases, xanthine, ascorbic acid, uric acid, oleic acid, and also some inorganic compounds used as food additives (Fe^2+^, Mn^2+^, Sn^2+^, SO_3_^2–^, S_2_O_5_^2–^, NO_2_^–^, etc.) [32,46]. In particular, fructose and glucose at concentrations of 1 g/L do not interfere [33], but at 25–100 g/L, an increase in a signal of 3–20% is observed. This effect becomes stronger with temperature rise [32]. Because of this, FCA requires that interfering substances should be removed first or the results should be adjusted to the interfering effect. Otherwise, FCA cannot be considered as a standard (reference) method for assessing the TPC, but it can be recommended for measuring antioxidant capacity [46,47]. The modified FCA allows for the simultaneous measurement of lipophilic and hydrophilic antioxidants at 665 nm in an isobutanol-aqueous medium with the addition of sodium hydroxide [48]. A novel spectrophotometric method with the use of Fast Blue BB diazonium salt was proposed to evaluate the TPC in food samples at alkaline pH and 420 nm [34]. Compared to FCA, higher values of TPC were found in most beverages, black rice, and quinoa, and lower values of the TPC were found in vitamin waters and flax seeds. Unfortunately, the obtained results were not supplemented with an interference study.

NIRS and FT-NIRS are used to detect the TPC in plant extracts [35,38], plant powders [36,39], and whole fruits [37,40]. These methods are based on the laborious development of a multivariate calibration model, which involves the analysis of a large number of similar samples and the use of chemometric tools for prognosis execution. However, once the model has been selected, a routine analysis can be completed in minutes or even in seconds. The indisputable advantages of NIRS and FT-NIRS are an indestructible analysis procedure and the absence of chemical reagents, while whole fruits are analyzed in real time (in situ) mode. However, infrared radiation has a small penetration depth, which is why these methods allow the detection of biologically active compounds only in the surface layers of the analyzed sample. Thus, the TPC is analyzed exclusively in the peel of whole apples [37,40]. Different types of fluorescence spectroscopy [41,42,43], including those coupled with chemometrics [43], are used to estimate the TPC in olive oil. Unfortunately, the developed NIRS, FT-NIRS, and fluorescence spectroscopy procedures were validated versus FCA, which, as was noted earlier, is not specific for phenolic compounds.

The use of HPLC requires complex and expensive equipment and skilled staff. This method is commonly used to separate, identify, and quantify phenolic compounds in extracts. After scanning the phenolic profile of a sample, the total content of phenolic acids, flavonoids, and phenols can be assessed by simply summing the concentrations of the main phenolic compounds of the respective class. Despite the fact that this approach has limitations due to the lack of data about unknown phenolic compounds in chromatographic libraries and a possible overlap of signals of some phenolic compounds with the same retention time, it was used for analyzing apricots [44] and coffee beans [45].

Biosensors based on phenol oxidases have been proposed as viable alternative analytical devices for the quantification of phenolic compounds. These biosensors were reviewed in [49,50,51,52,53,54,55,56,57,58,59]; however, they were not discussed in terms of the TPC estimation in food matrices. In order to denote the “pool“ of phenolic compounds determined by biosensors, terms such as “equivalent phenol content” [60], “total polyphenols concentration” [61], “polyphenol level” [62], “polyphenol index” [63,64,65,66], “bioelectrochemical polyphenol index” [66,67,68,69], “total polyphenol index” [69,70], “total phenol index” [70], and “phenolic antioxidant capacity” [71] are used along with the TPC. This paper introduces the unified term the “total phenolic index” (TPI) to denote the total concentration of phenolic compounds, which can be determined with the use of biosensor technologies. Thus, the purpose of this study was a comprehensive search and analysis of the publications where developed phenol oxidase-based biosensors were tested for assessing the TPI in food-related samples (FRSs), and the obtained results were validated using comparative methods.

## 2. Phenol Oxidases

Phenol oxidases, also known as polyphenol oxidases, are copper-containing enzymes that catalyze the oxidation of phenolic substrates in the presence of molecular oxygen as an electron acceptor [72]. The most well-studied (poly)phenol oxidases used in biosensors are laccase, tyrosinase, and catechol oxidase [49,50,51,52,53,54,55,56,57,58,59,72]. At a minimum, laccase and tyrosinase are able to take part in the oxidative modification of natural monophenols, such as coumaric, ferulic and syringic acids [54], guaiacol, eugenol, tyrosin, capsaicin, gingerol, carvacrol, thymol, sesamol, vanillin [73], and sinapinic acid. Therefore, the term “phenol oxidases” (POs) is more relevant than the term “polyphenol oxidases”. The catalytic properties of laccase and tyrosinase, which have been used in biosensors to assess the TPI in FRSs, are discussed below. Catechol oxidase (EC 1.10.3.1) is rarely used in biosensors [74,75] due to the ortho-phenol specificity of this enzyme [76]. However, the recent study [77] argued that *Taraxacum officinale* catechol oxidase unexpectedly exhibited tyrosinase activity, i.e., it was able not only to oxidize ortho-diphenols (catecholase activity), but also to ortho-oxygenate monophenols (cresolase activity). Apparently, the phenolic substrate specificity of catechol oxidase needs more detailed studies, which may further affect the use of this enzyme in biosensors. The physiological functions of POs are likely to vary depending on their origin. For example, plant POs are responsible for the enzymatic browning of fruits, which is associated with the oxidation of phenolic compounds and is observed when tissues are damaged or at the stage of postharvest storage [72].The substrate specificity and catalytic activity of POs can vary depending on their origin, isomorphism, and degree of purification, as well as on the medium parameters and immobilization method. Techniques for isolating POs from natural sources, including extraction, precipitation, dialysis, and lyophilization, can be critical to ensure the reproducibility of results. Commercial samples of laccase and tyrosinase are available, for example, from Creative Enzymes [78] and Sigma-Aldrich (part of Merck) [79].

### 2.1. Laccase

**Figure 2:**
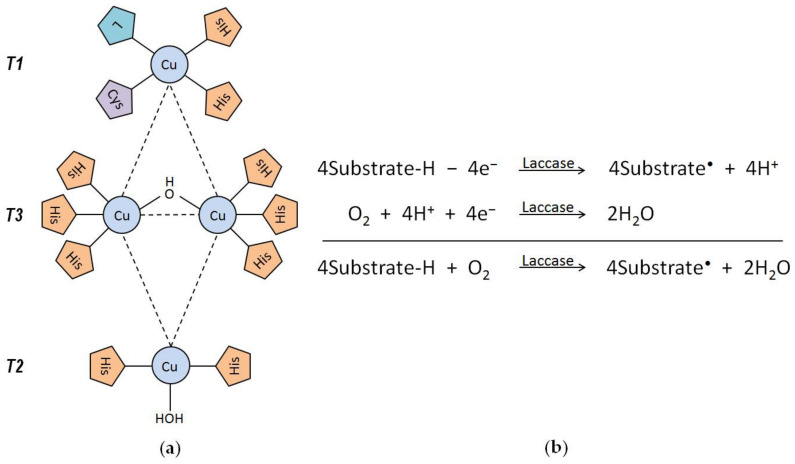
Schematic representation of the laccase catalytic site: His, histidine ligand; Cys, cysteine ligand; L, other ligand (**a**). Laccase catalyzed reactions (**b**) [80,81,82].

**Scheme 1:**
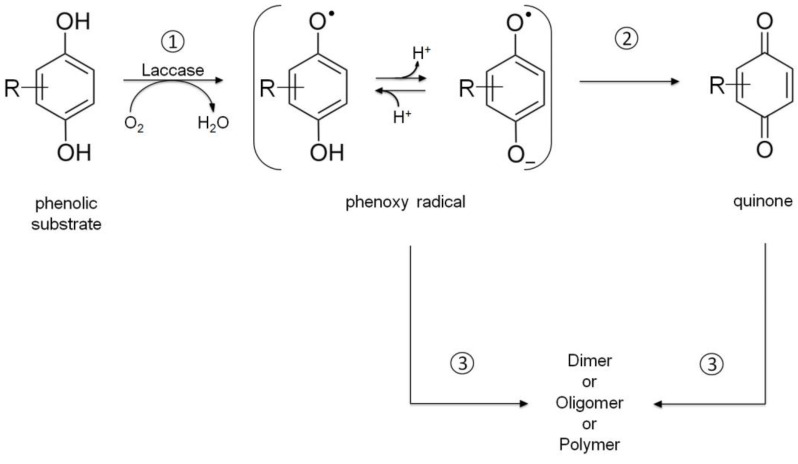
Phenolic substrate oxidation pathway catalyzed by laccase: enzymatic oxidation (1), non-enzymatic oxidation (2), and polymerization (3). Adapted from [82,84,85].

Laccases (EC 1.10.3.2) are multicopper oxidoreductases common in bacteria, fungi, and plants. They are oligomeric complexes of glycoproteins containing four copper atoms per monomer. The molecular weight of monomers can vary within the range of 50–130 kDa. The carbohydrate proportion includes mannose, galactose, and N-acetyl glucosamine [80,81,82].The active site of laccase contains four copper atoms that are distributed across three different sites (Figure 2a). The “blue“ site contains one copper atom of type I (T1) bound to four ligands, two of which are histidine, one is cysteine, and the fourth may vary depending on the source of the enzyme. The normal site includes one copper atom of type II (T2) bound to two histidine ligands, and the binuclear site combines two copper atoms of type III (T3), each bound to three histidine residues. The catalytic mechanism of laccase is due to the coordinated interaction between copper atoms in the active site and is based on the conjugation of the processes of one-electron oxidation of the substrate and the four-electron reduction of dioxygen (Figure 2b). In particular, the T1 copper atom is responsible for substrate oxidation, while the T2 and T3 copper atoms are combined into a trinuclear cluster, which is the site of oxygen reduction, as well as the formation and release of water [80,81,82]. In the native and reduced state of laccase, all copper atoms have an oxidation degree of +2 and +1, respectively [81,82,83]. Laccase is characterized by broad substrate specificity and catalyzes the oxidation of quite a number of physiological and non-physiological substances, such as phenols, aromatic amines, aromatic thiols, ascorbic acid, 2,2′-azino-bis(3-ethylbenzthiazoline-6-sulfonicacid) (ABTS), and potassium ferricyanide [80,82,83]. Scheme 1 illustrates the pathway of the phenolic substrate oxidation catalyzed by laccase in the presence of molecular oxygen [82,84,85]. First, phenolic compounds undergo one-electron enzymatic oxidation resulting in the formation of phenoxy radical. This phenoxy radical is an unstable intermediate that can be further non-enzymatically oxidized to quinone or polymerized to form the corresponding dimers, oligomers, and polymers. Some quinone products are able to autopolymerize. In addition to typical reactions given in Scheme 1, C_α_-carbonyl formation [84] is also possible in some particular cases.

### 2.2. Tyrosinase

**Figure 3:**
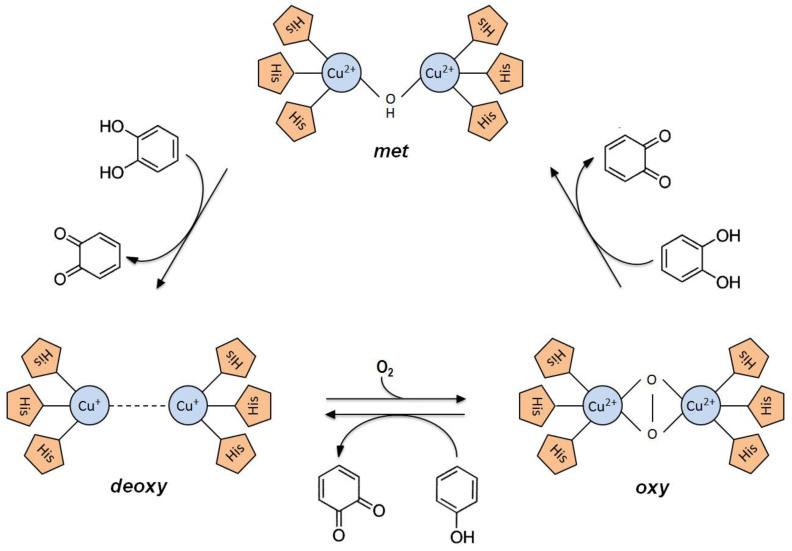
Schematic representation of the tyrosinase catalytic site and tyrosinase catalyzed reactions [87,88].

**Scheme 2:**
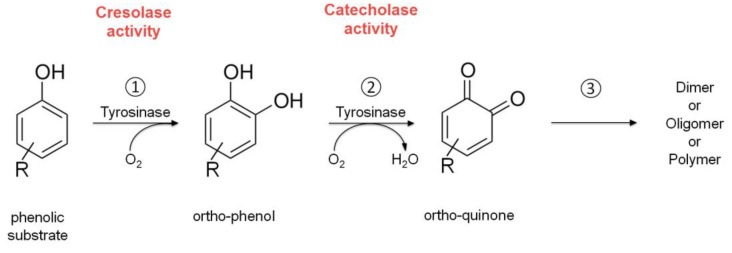
Phenolic substrate oxidation pathway catalyzed by tyrosinase: ortho-monooxygenation (1), oxidation (2), and polymerization (3). Adapted from [76,86,87].

Tyrosinases (EC 1.14.18.1) are dicopper oxidoreductases with various molecular weights. They are common in bacteria, fungi, plants, and some animals, including insects and mammals [76,86,87,88,89]. Tyrosinases belong to the type-3 copper protein family, as their active site contains two T3 copper commonly called CuA and CuB (Figure 3). To describe the catalytic properties of tyrosinase, various mechanisms have been proposed [76,87,88,89], which suggests the existence of different states of the active site, such as *met* [Cu(II)·Cu(II)], *deoxy* [Cu(I)·Cu(I)], and *oxy* [Cu(II)·O_2_·Cu(II)]. In particular, native *met*-tyrosinase and oxidized *oxy*-tyrosinase are responsible for binding and oxidizing phenolic substrates, while reduced *deoxy*-tyrosinase is responsible for binding and reducing oxygen. Tyrosinase has two types of activity [76,86,87,88]: it can oxygenate phenols in the ortho-position with regard to the hydroxyl group (cresolase or monophenolase activity) and can oxidize ortho-phenols into the corresponding ortho-quinones (catecholase or diphenolase activity). The resulting highly reactive ortho-quinone products are able to autopolymerize and form melanin-like polymers. The phenolic substrate oxidation pathway catalyzed by tyrosinase in the presence of molecular oxygen is shown in Scheme 2.

## 3. PO-Based Biosensors for Estimating TPI in FRSs

### 3.1. Definitions and Classification

According to the recommendations of the International Union of Pure and Applied Chemistry (IUPAC), a biosensor is understood as “a device that uses specific biochemical reactions mediated by isolated enzymes, immune systems, tissues, organelles or whole cells to detect chemical compounds usually by electrical, thermal or optical signals” [90]. For an electrochemical biosensor, the IUPAC Council proposed a more detailed definition: it “is a self-contained integrated device, which is capable of providing specific quantitative or semi-quantitative analytical information using a biological recognition element (biochemical receptor) which is retained in direct spatial contact with an electrochemical transduction element” [91]. A biosensor consists of two main components: (1) a biological recognition element, or biochemical receptor, that specifically interacts with an analyte; and (2) a transduction element, or transducer, that converts a biological signal into a measurable physical and chemical parameter.

Biosensors can be classified according to the type of biochemical receptor, transduction principle, analyte, or a combination of these factors. In the biosensors under discussion, POs serve as biochemical receptors that catalyze the oxidation of phenolic compounds in the presence of molecular oxygen as an electron acceptor. Depending on the number of POs, enzymatic and bienzymatic biosensors can be distinguished. Enzymatic biosensors are represented by laccase [60,61,62,63,64,66,68,70,71,92,93,94,95,96,97,98,99,100,101,102,103,104,105,106,107] and tyrosinase [61,65,66,67,70,107,108,109,110,111,112,113] based biosensors. Laccase and tyrosinase have different specificities for phenolic substrates; therefore, the combined immobilization of these two enzymes into a biosensor allows for the detection of more phenolic compounds [114,115], including meta-derivatives of phenolics [115]. Dual laccase-tyrosinase biosensors were used to evaluate TPI in beer [69], must, and wine [116]. Following the principle of transduction, PO-based biosensors for assessing TPI in FRSs can be divided into two groups: optical (Section 3.8) and electrochemical (Section 3.9). By the method of signal detection, optical biosensors are subdivided into fluorescent [92,93] and colorimetric [109]. Electrochemical biosensors can be subdivided into potentiometric [108], voltammetric [62,70,94,95,96,97,98,99,100,101,109], and amperometric [60,61,63,64,65,66,67,68,69,71,102,103,104,105,106,107,110,111,112,113,116].

### 3.2. POs Immobilization

**Figure 4:**
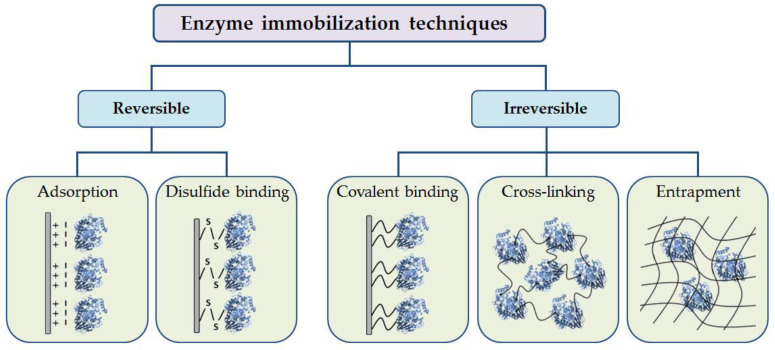
Common enzyme immobilization techniques.

The application of free enzymes in biosensors has many shortcomings. These might include the insufficient stability of an enzyme, the complex separation of an expensive enzyme from the analyzed solution, which makes it impossible to reuse it, etc. To overcome these limitations, immobilization is used, which is understood as the attachment of an enzyme to an insoluble carrier [51]. The attachment of an enzyme to a nanostructured carrier has recently been defined as “nano-immobilization” [55]. The most common enzyme immobilization methods are adsorption, covalent binding, cross-linking, and entrapment (Figure 4). Reversible and irreversible types of immobilization are determined by the nature of the enzyme-to-carrier binding [117].

Adsorption techniques are based on the formation of ionic, hydrogen, Van der Waals bonds, and other physical interactions between the enzyme and the carrier, which can be broken under operating conditions or during storage of the biosensor [51,55,117]. Due to this fact, the use of adsorption as an independent method of immobilization has recently been quite limited [105].

Covalent binding techniques are based on the formation of covalent bonds between the functional groups of the enzyme and the reactive groups of the activated carrier. Carboxylated multi-walled carbon nanotubes (cMWCNTs) [62,95,96,97] and carboxylated polyvinyl chloride (cPVC) [108] were used to obtain reactive COOH-groups, while chitosan [62], polyaniline (PANI) [62,95,96,97], aminothiols [94,98], and (3-aminopropyl)triethoxysilane (APTES) [113,116] served as sources of reactive NH_2_-groups. Cysteamine [94], 4-mercaptoaniline [98], and other aminothiols may cause the formation of self-assembled monolayers with terminal NH_2_-groups on the surface of a gold electrode. The subsequent covalent addition of POs occurred with the help of chemical reagents, but it was also possible without their participation. Thus, N-(3-dimethylaminopropyl)-N’-ethylcarbodiimide hydrochloride (EDC) was able to build amide (–CO–NH–) bonds between reactive COOH-groups of the electrode and exposed NH_2_-groups of the enzyme [108], while glutaraldehyde (GA) was able to form imine (–CH=N–) bonds between reactive NH_2_-groups of the electrode and exposed NH_2_-groups of the enzyme [62,94]. Covalent binding, as a rule, is generally irreversible. However, disulfide (–S–S–) bonds could be broken in a medium with a high content of low molecular weight thiols [117].

Cross-linking techniques are based on the formation of intermolecular bonds between enzyme molecules, using bi-functional reagents. GA is well-known as a cross-linking agent that ensures the formation of multi-point covalent linkages between laccase and tyrosinase molecules [63,66,67,69,70,98,106,109,111]. In the enzyme molecules, GA was primarily reactive towards NH_2_-groups of amino acids and the SH-group of cysteine [118]; moreover, the best binding irreversibility was observed within the pH range from 7.0 to 9.0 [119]. The addition of bovine serum albumin (BSA) to GA was used to form cross-links and aimed at increasing the formation effectiveness and stability of cross-linked enzyme aggregates [61,107,113].

Entrapment techniques are based on the inclusion of enzyme molecules in a paste or polymer matrix. In the fabrication of biosensors, laccase [99,100,101] and tyrosinase [107] were introduced directly into an electrically conductive paste. To capture and hold POs, various synthetic and natural polymers were used, such as polyvinyl alcohol azide-unit pendant water-soluble photopolymer (PVA-AWP) [60], chitosan [61,110], polyazetidine prepolymer (PAP) [64], poly(3,4-ethylenedioxythiophene) (PEDOT) [65], ammonia-neutralized Nafion [71,104], chitosan-galactomannan composite [98], polypyrrole (PPy) [102], kappa-carrageenan [112], and diglycerysilane [116]. To manufacture conductive (PANI, PEDOT, PPy) and photosensitive (PVA-AWP) polymers, electropolymerization and photocuring techniques were used, respectively. It was earlier reported that laccase immobilization can improve biosensor performance [51] and change the specificity of this enzyme with respect to phenolic substrates [55], which is why it is recommended to test several methods of immobilization in order to select the optimal one.

Some novel methods of enzyme immobilization, such as matrix assisted pulsed laser evaporation (MAPLE) [103], cold plasma polymerization [120], electrospray deposition [121], laser printing [122], and piezoelectric inkjet printing [123], are high-tech and effective; however, they have not yet been widely used in biosensors.

### 3.3. Functions of Nanomaterials

**Figure 5:**
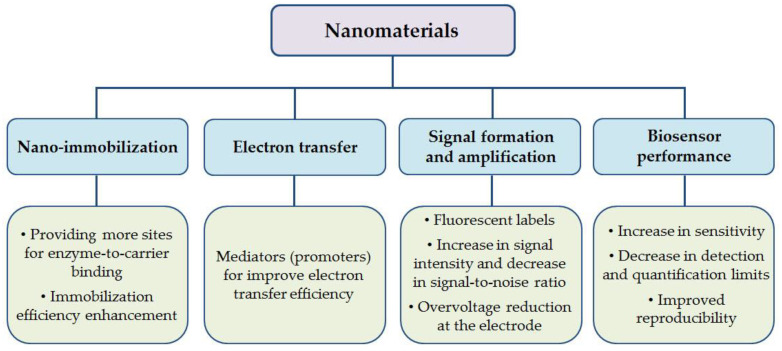
Functions of nanomaterials in PO-based biosensors.

Nanomaterials have a high surface-to-volume ratio and a unique set of electrical, magnetic, optical, chemical, and other properties, which allows them to be used as functional components in (bio)sensors. The main functions of nanomaterials in PO-based biosensors include nano-immobilization, electron transfer, signal formation, and amplification, improving the biosensor performance (Figure 5). Bioconjugates of negatively charged laccase with positively charged gold nanoparticles (AuNPs) [92] and carbon dots (CDs) [93] were used in fluorescent biosensor technologies. Single nanoparticles, binary nanocomposites, and nanohybrids were used in electrochemical biosensors. Single-walled carbon nanotubes (SWCNTs) [64], multi-walled carbon nanotubes (MWCNTs) [64,101], AuNPs [67,102,109,111], and carbon black [107] were used to improve enzymatic electrodes performance. Synergetic electrocatalytic effects were obtained applying cMWCNTs combined with nanoparticles of copper (CuNPs) [62], silver (AgNPs) [96], nickel (NiNPs) [97], and iron (II,III) oxide (Fe_3_O_4_NPs) [95]; reduced graphene oxide (rGO) combined with MWCNTs [61] or platinum nanoparticles (PtNPs) [104]; graphene nanoplatelets (GNPLs) combined with AuNPs [71]; and combinations of silver and zinc oxide nanoparticles (AgNPs and ZnONPs) [94], molybdenum disulfide nanoflakes, and graphene quantum dots (MoS_2_NFs and GQD) [105]. A novel reduced graphene oxide-glycolchitosan (rGO-GCS) nanohybrid was used for the assembly of an amperometric laccase biosensor [106].

### 3.4. Catalytic Cycle

**Figure 6:**
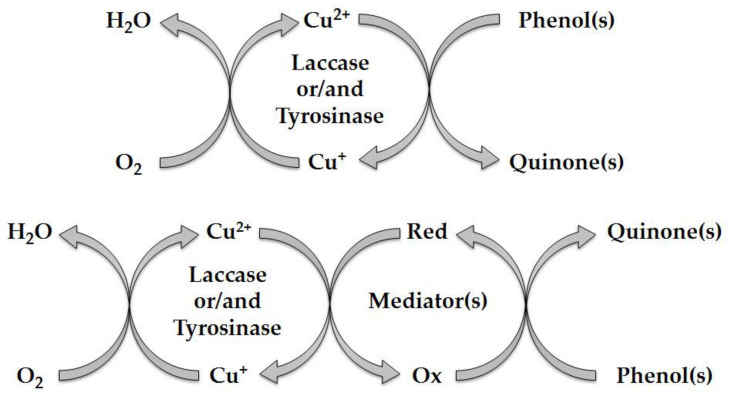
Schematic representation of catalytic cycles implemented in PO-based biosensors: independent of a mediator (**above**) and dependent of a mediator (**below**).

In enzymatic biosensors, a catalytic redox process occurs and it involves an enzyme (laccase or tyrosinase), a substrate (phenolic compound), and an electron acceptor (molecular oxygen). The copper-containing active site of the enzyme catalyzes the oxidation of the phenolic substrate and the reduction of oxygen. The product of the phenol enzymatic oxidation cannot be always precisely identified, but it is generally accepted to be quinone. In this case, oxygen is reduced to water. These redox processes could proceed with the participation of one or two mediators, which might be subphthalocyanine [70], ferrocene [113], or various nanomaterials (Section 3.3, Section 3.8 and Section 3.9). The enzyme and quinone revive to the initial oxidation state chemically (with phenol or other reducing agent) or electrochemically (at the electrode at the appropriate potential) and then react again. Thus, a catalytic cycle is formed in which the phenol substrate is reused, which contributes to the increased sensitivity of the biosensor [49,50,55,61,62,64,68,92,96,102,104,105,107,109,111,113]. In bienzymatic biosensors, apparently, the simultaneous oxidation of two phenolic substrates can occur as a result of the presence of both laccase and tyrosinase. The generalized schemes of catalytic cycles implemented in PO-based biosensors are shown in Figure 6.

### 3.5. TPI Estimation

The accuracy of the TPI assessment with the use of PO-based biosensors is affected by some factors, such as the choice of the standard, the analytical procedure used, interference, and validation. The TPI assessment is carried out by using reference phenolic compounds (standards). The most frequently used are gallic and caffeic acids. In this case, the oxidation of gallic acid in laccase [60,61,63,66,92,104,107,116] and laccase-tyrosinase [69,116] biosensors occurs with low kinetics and is characterized by low sensitivity. Gallic acid was reported to show inhibitory behavior on laccase activity [124]; hence, gallic acid was not recommended to be used as a standard in laccase-containing biosensors. Calibration and standard addition methods can be applied for TPI estimation. The calibration method implies the interpolation of a sample signal to a standard calibration curve, which is obtained under optimum operating conditions. However, this method does not take into account matrix effects; therefore, it could lead to biased results. There are two approaches to reducing the impact of matrix effects in the external calibration method. The first approach is to use a sample at the highest possible dilution for the analysis. The second approach is to use a phenolic compound that prevails in the content in the analyzed sample as a standard. Information on the content of phenolic compounds in FRSs can be obtained from the Phenol-Explorer electronic database [125], which currently contains data about over 400 products. For instance, chlorogenic acid and tyrosol may be relevant standards for coffee and olive oil, respectively, while catechin is the most commercially available standard for tea and wine [125]. On the contrary, the standard addition method can be the calibration method of choice when matrix effects appear. The IUPAC-recommended procedure involves adding increasing amounts of the standard to the sample and then extrapolating the resulting calibration curve to the negative semiaxis [126]. Flow injection analysis (FIA) systems provide well-defined hydrodynamic measurement conditions, can be used for automated sample processing, and are convenient in evaluating analytical characteristics of biosensors [91]. The value of the TPI depends on FIA conditions [63], the source of an enzyme [64], and the pH of the medium. At the same time, the pH of the medium affects not only the enzymatic activity, but also the ratio of free and bound polyphenols in the analyzed sample (Section 3.7).

### 3.6. Interference

Signal alteration in laccase-based biosensors was recorded in the presence of many non-phenolic compounds (Table 1), of which ascorbic acid, citric acid, fructose, and glucose showed interference effects independent of the principle of biosensor transduction. It was reported that ascorbic acid was a non-phenolic substrate of laccase [80,83], while citric acid was able to chelate copper ions in the active site of laccase and act as an inhibitor [127]. The mechanism of carbohydrate interference in biosensors based on purified POs still remains unclear. In the case of unpurified and partially purified POs, carbohydrates could serve as a nutrient medium (sources of carbon) and promote the production of additional amounts of both laccase [128] and tyrosinase [129]. Laccase is not specific for phenolic compounds and, in addition to ascorbic acid, also oxidizes aromatic amines and thiols [80,82,83], which may be present in some beverages, including wine, beer, and coffee [130,131,132]. Compared to laccase, tyrosinase has catalytic specificity for phenolic compounds; however, potentiometric [108] and amperometric [110] tyrosinase biosensors could still suffer from interference from ascorbic acid. The interfering effect of ascorbic acid in tyrosinase biosensors may be due to its ability to reduce the induction period of the hydroxylation reaction [133] or to inhibit tyrosinase by copper chelation [134]. The studies of interference in laccase-tyrosinase biosensors are limited, which offers new research opportunities. Only the interference of potassium metabisulfite in an amperometric laccase-tyrosinase based biosensor was reported [116]. Since ascorbic acid (vitamin C) can be found in many foodstuffs as a natural metabolite or food additive (E300–E302) [135,136], the preliminary removal of ascorbic acid from the analyzed sample can be considered as a general strategy aimed at enhancing specificity and/or analysis accuracy in laccase, tyrosinase, and laccase-tyrosinase biosensors.

### 3.7. Validation

Validation of the TPI assessment is carried out using the following comparative methods: FCA [60,62,63,64,66,67,68,69,92,93,94,95,96,97,98,100,101,102,103,104,105,106,107,108,111,112,116], HPLC [109], antioxidant capacity assays with the use of ABTS (2,2′-azino-bis(3-ethylbenzthiazoline-6-sulfonic acid)) [61,65,68,71], and DPPH (2,2-diphenyl-1-picrylhydrazyl) [99,101]. In the analysis of wines, the spectrophotometric polyphenol index (I_280_) can additionally be used [116], which is determined following the method described in [137]. As a rule, a high positive correlation or statistically insignificant differences between the obtained results serve as a criterion for successful validation.

**Figure 7:**
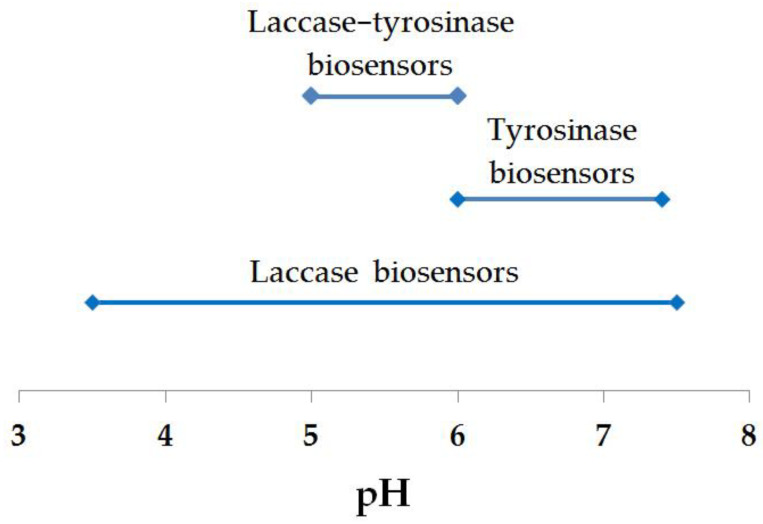
Operating pH ranges of PO-based biosensors.

The discrepancy between the results obtained using a biosensor and FCA may be due not only to the difference in oxidative capacity between the enzyme and the Folin-Ciocalteu reagent, but also due to the difference in pH and its effect on the ratio of free vs. bound polyphenols. The complex formation of polyphenols with most metal cations was reported to occur at the pH range from 2 to 8 [138,139,140], while the aggregation of proteins with tannins occurred at pH values close to the isoelectric point (pI) [141,142]. At the same time, the plant proteome consisted mainly of acidic proteins with an average value of pI ≈ 5.62 [143]. Since pH ≈ 10 was used in FCA [32], and PO-based biosensors function in the pH range from 3.5 to 7.5 (Figure 7), the discussed interactions of polyphenols with metal cations and proteins could be stronger in acidic and neutral media and might lead to the underestimation of analytical results. In order to ensure the least divergence of results and to perform an improved and more careful validation, the analysis with the use of PO-based biosensors must be carried out in alkaline or weakly alkaline media. In this case, most fungal laccases would be limited, since they exhibited maximum activity with regard to phenolic substrates in the pH range from 3.0 to 5.5 and became practically inactive in neutral and alkaline media [144,145]. Recently, the appearance of the first fungal laccase with an alkaline pH optimum was reported [146], which could hold considerable promise for being used in biosensors.

### 3.8. Optical Biosensors

#### 3.8.1. Fluorescent Biosensors

Fluorescence is the emission of light, occurring when molecules of some substances absorb the energy of excitation. In the fluorimetric analysis, the measured signal is the intensity of fluorescence, observed when fluorescence-active compounds (fluorophores) are irradiated with a monochromatic radiation source. Andreu-Navarro et al. [92] discussed a long-wavelength fluorimetric method for the determination of TPI, which involved the use of laccase from *Trametes versicolor*, long-wavelength fluorophore indocyanine green, and positively charged AuNPs. A stopped-flow mixing device was applied for the automatic and fast mixing of reagents. The measurements were carried out at pH 7.5, when laccase has a negative charge. Compared to negatively charged AuNPs obtained by the Turkevich method, positively charged AuNPs obtained by the Caruso method changed the kinetics of the system. The observed increase in the induction time was caused by the electrostatic interaction between negatively charged laccase and positively charged AuNPs. The dye was oxidized by laccase, which led to a rapid decrease in its fluorescence. This process was slowed down in the presence of phenolic compounds, giving a time period directly proportional to the concentration of added polyphenols. The limits of detection (LODs) for catechol and hydroquinone were 0.01 μM. The authors reported that the use of long-wavelength fluorimetry allowed them to avoid potential signals from the sample matrix that can appear at lower wavelengths. In particular, ascorbic acid at a concentration of 2 μM did not interfere. However, the consumption of the enzyme was higher than in immobilization-based biosensor methods. An enzymatic fluorimetric method was used to evaluate TPI in commercial beverages, and the results are comparable to those of FCA.

Mediavilla et al. [93] synthesized Lac–CD bioconjugate, which combines the specificity of laccase from *Trametes versicolor* (Lac) to phenolic compounds and the optical properties of carbon nanodots (CDs). Presumably, the conjugation process was based on the electrostatic interaction between negatively charged laccase and positively charged CDs, with a zeta potential of +90 mV. The resulting bioconjugate was used as a fluorescent label to track the oxidation reaction of gallic acid under model conditions and phenolic compounds in FRSs. The LOD for gallic acid was found to be 7.4 μM. Ascorbic acid, citric acid, fructose, glucose, and sodium sulfite were tested as potentially interfering compounds. These compounds had no effect on the fluorescent signal when they were present in the solution with a 100-fold lower concentration than gallic acid. When these compounds were present in the solution with a 10-fold lower concentration than gallic acid, the fluorescent signal increased by 5–10%. The results obtained from the FRS analysis were compared with the FCA results.

#### 3.8.2. Colorimetric Biosensors

Datta et al. [109] developed a colorimetric biosensor by immobilizing mushroom tyrosinase on egg shell membrane. The resulting biosensor showed a change in color while contacting with standard solutions (gallic acid, caffeic acid, and catechin hydrate) and real samples (wine, tea). However, the biosensor capabilities were limited by a qualitative assessment of the shade scale only. Due to the simplicity and rapidity of the analysis, the authors reported on the possibility of using the developed biosensor in the field. The use of digital image processing might result in a quantitative evaluation.

### 3.9. Electrochemical Biosensors

In comparison with non-enzyme electrodes, enzyme electrodes have some advantages due to a lower potential of polyphenol electrooxidation, which reduces the likelihood of the interference of electroactive substances and quinones polymerization, whose resulting products are responsible for the contamination of the electrode surface [49,115]. Table 2 presents electrochemical PO-based biosensors for assessing TPI in FRSs. They are grouped by the type of enzyme and detection method. On the basis of a signal detection method used, biosensors are divided into potentiometric, voltammetric, and amperometric. The use of various modifiers offers good opportunities for the development of biosensors based on electrochemical transducers. Gold electrodes [62,94,95,96,97,98,110], electrodes with a platinum anode [68,112], indium tin oxide (ITO) coated glass slides [70], screen-printed carbon electrodes (SPCEs) [60,64,71,102,103,104,105,111,113,116], glassy carbon electrodes (GCEs) [61,63,67,106,109], sonogel-carbon electrodes (SCEs) [65,66,69], and carbon paste electrodes (CPEs) [99,100,101,107] were used for fabricating electrochemical PO-based biosensors for assessing TPI in FRSs. The list of carbon-containing electrodes could be supplemented by carbon cloth and carbon paper, which are widely used in the development of potentiometric [147], voltammetric, and amperometric [148] (bio)sensors. Commercially available disposable SPCEs have a miniature planar configuration that enables them to perform electrochemical measurements in the single-drop mode [102,103,104,105,111]. A short storage lifetime could be a significant disadvantage of electrochemical biosensors that limits their use in the laboratory and their commercialization. As a rule, the longer storage time of PO-based biosensors may lead to a progressive decrease in the electroanalytical response, which might be due to a gradual decrease in enzyme activity or its denaturation. The studies [61,66] argued that, compared to laccase biosensors, tyrosinase biosensors had lower stability in the long run. The use of new immobilization methods (Section 3.2) could improve the performance of electrochemical PO-based biosensors, including their long-term stability.

#### 3.9.1. Potentiometric Biosensors

In potentiometry, the measured signal is the potential difference between the non-polarizable working electrode and the reference electrode. Draghi et al. [108] developed a label-free potentiometric biosensor by covalently immobilizing tyrosinase derived from a crude banana peel (*Musa acuminata*) extract onto the surface of a two-layer cPVC-based solid-contact transducer. The tyrosinase enzyme was immobilized by covalent bond on the upper layer of the transducer with the use of EDC, which activated the COOH-groups of cPVC. The potentiometric biosensor method proved to be faster than FCA.

#### 3.9.2. Voltammetric Biosensors

In voltammetric methods, the measured signal is the current flowing through the working electrode and depends on the changing potential applied to it. Pundir et al. developed several biosensors based on a gold electrode modified with nanocomposites and covalently immobilized laccase extracted from *Ganoderma* sp. Rckk02, which were characterized by long-term stability. The immobilization of laccase by covalent bond was carried out using GA [62,94] or without any cross-linking agent [95,96,97]. For the latter, cMWCNTs–PANI hybrid film was formed on the surface of a gold electrode by the electropolymerization of aniline. Fe_3_O_4_NPs, AgNPs, or NiNPs were electrodeposited onto the film surface. At the final stage, laccase was applied onto the electrode by drop-coating and was kept for 12 h before washing. The key role in the immobilization process was played by the COOH-groups of cMWCNTs. They formed amide bonds with the NH_2_-groups of laccase and the NH_2_-groups of PANI, which was confirmed by the FT-NIRS data. It was suggested that the electrocatalytic properties of laccase biosensors improved when the following binary nanocomposites were used: cMWCNTs and CuNPs [62], AgNPs and ZnONPs [94], cMWCNTs and Fe_3_O_4_NPs [95], cMWCNTs and AgNPs [96], cMWCNTs and NiNPs [97].

Gonzalez-Anton et al. [70] studied the electron mediator properties of hexa-chloro boron subphthalocyanine (ClSubPc), hexa-phenoxy boron subphthalocyanine (PhOSubPc), and tri-*tert*-butyl boron subphthalocyanine (*t*-BuSubPc) in ITO-based laccase and tyrosinase biosensors. The immobilization of *Trametes versicolor* laccase and mushroom tyrosinase was carried out by GA cross-linking. The presence of subphthalocyanines in PO-based biosensors was characterized by the improvement of the charge transfer rates and the LODs by one order of magnitude, reaching approximate 10^–7^ M values. However, after the biosensors were removed from the solution, a noticeable decrease in the intensity of voltammetric peaks was observed; therefore, the biosensors are meant to be used only once.

Boubezari et al. [98] reported a laccase biosensor with a record low LOD. Its value for pyrocatechol was 10^–16^ M. The fabrication of this biosensor included several steps: (1) the formation of the 4-aminothiophenol self-assembling monolayer on the surface of a gold electrode; (2) the preparation of a biorecognition element by encapsulating *Trametes versicolor* laccase in a chitosan–galactomannan composite; (3) the drop-coating of the biorecognition element on the gold electrode; and (4) cross-linking in the GA vapor.

De Souza Gil et al. developed a series of biosensors using CPE, in which the laccase crude extract of *Pycnoporus sanguineus* was directly introduced into an electrically conductive paste. Different types of pastes were used: regular graphite paste [99]; graphite paste modified with organo-functionalized silica [100], MWCNTs, and deoxyribonucleic acid (DNA) [101]. For the latter, MWCNTs and DNA contributed to a stronger response and a lower overvoltage of the enzyme electrode, respectively.

Datta et al. [109] immobilized mushroom tyrosinase on the surface of an egg shell membrane modified with AuNPs. This membrane was placed onto the GCE with the help of an ‘O’ ring, and the resulting construction served as a voltammetric biosensor. The voltammetric response was higher in the presence of AuNPs, which was due to their ability to improve the electron transfer rate in the enzymatic reaction.

#### 3.9.3. Amperometric Biosensors

Amperometric biosensors are most widely represented among PO-based biosensors. In amperometry, the measured signal is the current flowing through the working electrode at a constantly applied potential. Modern equipment enables us to take amperometric measurements in hydrostatic, hydrodynamic, and FIA conditions. Vlamidis et al. [61] developed laccase and tyrosinase biosensors based on GCEs modified with the binary nanocomposite rGO and MWCNTs. A variety of methods has been tested in the immobilization of enzymes. The GA and BSA system proved to be the best for the *Trametes versicolor* laccase immobilization, while chitosan showed good performance in the mushroom tyrosinase immobilization. The amperometric response of the laccase biosensor was studied with regard to the sequence of carbon nanomaterials deposition. The highest sensitivity during catechol oxidation was observed in the case of a hybrid configuration of nanomaterials obtained as a result of the co-application of rGO and MWCNTs.

Pingarrón et al. developed biosensors based on GCEs using *Trametes versicolor* laccase [63,106] or mushroom tyrosinase [67] as a biological recognition element. POs were immobilized by GA cross-linking. In [63], a laccase biosensor was used under both batch and FIA conditions with amperometric detection. The FIA mode resulted in lower LODs for gallic acid and caffeic acid and an increase in TPI compared to the batch mode. The study [106] reported a novel nanohybrid rGO-GCS, which was obtained by covalent cross-linking with glycol chitosan (GCS) onto the surface of graphene oxide (GO). The components of the nanohybrid performed different functions in the laccase biosensor. GCS ensured the loading of the enzyme onto the electrode and presumably contributed to the preservation of its active conformation after immobilization due to the presence of the NH_2_-groups and hydrophilic properties, respectively. rGO improved the electrocatalytic properties of the biosensor. In [67], a tyrosinase biosensor was obtained by enzyme immobilization on the GCE modified with electrodeposited AuNPs. Electrocatalytical properties of the enzyme electrode, modified with AuNPs, were significantly better than those of the non-modified.

Hidalgo-Hidalgo de Cisneros et al. developed an SCE, which was used as a transducer in laccase [66], tyrosinase [65], and laccase-tyrosinase [69] biosensors with amperometric detection. In [66], *Trametes versicolor* laccase or mushroom tyrosinase was immobilized on a SCE with the use of the GA and Nafion system. After comparing the stability, reproducibility, and accuracy of enzymatic electrodes, the laccase-based biosensor was chosen as the best for evaluating TPI in real beer samples. García-Guzmán et al. [65] immobilized mushroom tyrosinase in a PEDOT matrix during the electropolymerization of the precursor monomer on SCE, using a new sinusoidal current electrodeposition technique that improved the loading of the enzyme onto the electrode.

Zrinski et al. [71] prepared an SPCE with the use of carbon paste modified with GNPLs. The AuNP suspension and a cocktail containing *Rhus vernicifera* laccase and ammonia-neutralized Nafion were sequentially drop cast on the electrode. Nanomaterials considerably improved the kinetics of the electron transfer in the laccase biosensor, which was observed in the hydroquinone cyclic voltammogram as a shift of the anodic/cathodic peaks to the region of lower/higher potentials with a simultaneous increase in the intensity of these peaks. An important analytical consequence of the use of nanomaterials was the better reproducibility of the amperometric laccase biosensor. It was concluded that the GNPL and AuNP nanocomposite was a supporting matrix for laccase immobilization and a suitable matrix for the detection of phenolic compounds.

Mohtar et al. [102] described a laccase biosensor, whose manufacturing scheme is based on the AuNP electrodeposition on the surface of an SPCE, the dropwise deposition of an enzymatic layer, and the formation of a PPy film through the electropolymerization of the precursor monomer. The electrodeposition of AuNPs enlarged the effective area for laccase immobilization and improved the electrocatalytic properties of the biosensor. The laccase biosensor had a shorter response time than the FCA.

Verrastro et al. [103] proposed an amperometric biosensor based on *Trametes versicolor* laccase immobilized onto an SPCE using the innovative MAPLE (matrix assisted pulsed laser evaporation) technique. In MAPLE, pulsed laser radiation was focused inside a vacuum chamber and aimed at a rotating target, which was a frozen enzymatic solution. The energy of the laser pulse was absorbed mainly by the solvent and converted into thermal energy. As a result, the solvent evaporated, and the enzyme molecules were deposited as a thin film on the relevant substrate. The obtained laccase biosensor demonstrated good sensitivity, linearity, selectivity, stability, and lifetime.

Eremia et al. [104,105] discussed two amperometric biosensors based on an SPCE modified with nanomaterials and laccase from *Trametes versicolor*. In [104], rGO, PtNPs, and laccase were sequentially drop-coated onto the SPCE. It was noted that rGO and PtNPs nanocomposite had a synergistic effect on electron transfer and enlarged the electroactive surface area of the laccase biosensor. In [105], MoS_2_NFs, GQDs, and laccase were also sequentially drop-coated onto the SPCE, but the enzymatic layer was additionally stabilized by the embedment of Nafion neutralized with ammonia. MoS_2_NF and GQD nanomaterials provided excellent electronic conductivity and reduced the charge transfer resistance in the laccase biosensor. As a result, the electrochemical response of caffeic acid was characterized by a shift of the anodic/cathodic peaks to the region of lower/higher potentials, with a simultaneous increase in the intensity of these peaks.

Nadifiyine et al. [107] proposed laccase and tyrosinase biosensors based on a carbon black paste electrode (CBPE). Laccase from *Trametes versicolor* and tyrosinase from *Agaricus bisporus* were immobilized by cross-linking with the GA and BSA system. In the amperometric determination of catechol, the CBPE-based tyrosinase biosensor demonstrated higher sensitivity, lower LOD, and a wider linear range compared to the graphite paste electrode-based tyrosinase biosensor. The obtained results allowed the authors to conclude that carbon black nanomaterial had more attractive properties for paste-electrode fabrication.

Montereali et al. fabricated tyrosinase [113] and laccase-tyrosinase [116] biosensors using SPCE as a transducer and ferrocene as a mediator. The SPCE was prepared using graphite paste modified with ferrocene. The ferrocene-modified electrode was activated for one hour in an APTES solution in order to form functional NH_2_-groups on its surface. The tyrosinase biosensor was developed by cross-linking mushroom tyrosinase with the BSA and GA system. The laccase-tyrosinase biosensor was developed by the co-immobilization of *Trametes versicolor* laccase and mushroom tyrosinase into a sol-gel matrix of diglycerysilane. The bienzymatic biosensor showed higher LODs for some phenolic substrates (caffeic acid, catechin, and catechol) in comparison to laccase and tyrosinase biosensors. However, this small loss of LODs in the bienzymatic biosensor was compensated for when assessing TPI in a sample containing a large number of phenolic compounds.

## 4. Conclusions and Future Perspectives

PO-based biosensors are used in the quantification of phenolic compounds. They have several advantages over traditional analytical methods, such as minimal sample preparation, faster response time, reproducibility, relatively low cost, simple analysis procedure, and the ability to miniaturize and automate. However, the effectiveness of PO-based biosensors in the analysis of food samples, which are complex multicomponent matrices, has not been considered in detail in previous reviews. This review highlights the catalytic properties of laccase and tyrosinase and focused on enzymatic and bienzymatic biosensors based on laccase and tyrosinase, which are used for assessing TPI in FRSs. Nanomaterials are involved in the processes of immobilization, electron transfer, signal formation, and amplification, and they improve the performance of PO-based biosensors. Fluorescent biosensor technologies are based on the use of nanobioconjugates. Better characteristics of electrochemical biosensors are ensured by using single nanoparticles, binary nanocomposites, or nanohybrids. In the future, nanomaterials and digital image processing should be applied in colorimetric biosensors. The accuracy of the TPI estimation with PO-based biosensors depends on several factors, such as the choice of the standard, the analytical procedure used, interference, and validation. Since laccase is not a phenolic-specific enzyme, laccase biosensors suffer from interference. Tyrosinase is an enzyme specific for phenolic compounds, but tyrosinase electrochemical biosensors have lower long-term stability compared to laccase-based biosensors. Laccase-tyrosinase biosensors make it possible to detect a larger number of phenolic compounds; however, interference effects have not been fully studied. Strategies for reducing interference in PO-based biosensors may include: (i) removing ascorbic acid, which is a non-phenolic laccase substrate and tyrosinase inhibitor, from the test sample; (ii) using highly purified enzymes in the biosensor construction in order to reduce the interfering effect of carbohydrates. Further research should encourage the use of new immobilization techniques, such as matrix assisted pulsed laser evaporation, cold plasma polymerization, electrospray deposition, laser printing, and piezoelectric ink jet printing. These immobilization techniques might contribute to a better performance of PO-based biosensors. Another research avenue might be the search for new POs that are specific for phenolic compounds and could retain their catalytic activity for a long time after immobilization.

## Figures and Tables

**Table 1:** Interference in laccase based biosensors.

Interferant	Standard: Interferant	Signal Change (%)	Detection Method	Source
Acetic acid	1:1	14–20	Cyclic voltammetry	[94,96]
Ascorbic acid	1:0.1 1:1 1:0.05 NR 1:1 NR 1:10,000	7 10–15 9 NR 5 NR 17	Fluorimetry Cyclic voltammetry Cyclic voltammetry Differential pulse voltammetry Amperometry Amperometry Amperometry	[93] [94,96] [97] [99] [71] [103] [106]
Caffeine	1:1	23	Amperometry	[71]
Citric acid	1:0.1 1:1	8 5–11	Fluorimetry Cyclic voltammetry	[93] [94,96,97]
Cysteine	1:1 1:0.002	12–15 9	Cyclic voltammetry Cyclic voltammetry	[94,96] [97]
Ethanol	1:1	13	Cyclic voltammetry	[96]
Fructose	1:0.1 1:1 1:0.6	7 9–26 6	Fluorimetry Cyclic voltammetry Cyclic voltammetry	[93] [94,96] [97]
Glucose	1:0.1 1:1 1:0.22 1:1	5 10–24 10 5	Fluorimetry Cyclic voltammetry Cyclic voltammetry Amperometry	[93] [94,96] [97] [71]
Sodium sulfite	1:0.1	10	Fluorimetry	[93]

Standard, reference phenolic compound; NR, not reported.

**Table 2:** Electrochemical PO-based biosensors for assessment TPI in FRSs.

Working Electrode ^1^	PO Source ^2^	Immobilization Technique ^3^	Detection Method ^4^	LOD (μM)	Storage Stability ^5^	FRSs	Validation ^6^	**Ref.**
**Laccase Biosensors**								
Lac/ClSubPc/ITO	TV	Cross-linking using GA	CV	0.169	NR	Wine	NR	[70]
Lac/PhOSubPc/ITO	TV	Cross-linking using GA	CV	0.155	NR	Wine	NR	[70]
Lac/*t*-BuSubPc/ITO	TV	Cross-linking using GA	CV	0.485	NR	Wine	NR	[70]
Lac/CuNPs-CS/cMWCNTs-PANI/Au	GsR	Covalent binding with GA	CV	0.156	−20%/7 m	Tea and alcoholic beverages	FCA	[62]
Lac/CA/AgNPs/ZnONPs/Au	GsR	Covalent binding with GA	CV	0.05	−25%/5 m	Wine	FCA	[94]
Lac/Fe_3_O_4_NPs/cMWCNTs-PANI/Au	GsR	Covalent binding	CV	0.03	−25%/4 m	Tea	FCA	[95]
Lac/AgNPs/cMWCNTs-PANI/Au	GsR	Covalent binding	CV	0.05	−20%/4 m	Tea and alcoholic beverages	FCA	[96]
Lac/NiNPs/cMWCNTs-PANI/Au	GsR	Covalent binding	CV	0.05	−15%/4 m	Fruit juices	FCA	[97]
Lac-CS-GAL/4ATP/Au	TV	Entrapment in CS-GAL,cross-linking using GA	CV	10^–10^	15 d	Olive oil	FCA	[98]
Lac-CPE	PS	Incorporation into the paste	DPV	0.01	NR	Fruits dry extracts	DPPH	[99]
Lac-CPE	PS	Incorporation into the paste	DPV	NR	NR	Honey	FCA	[100]
Lac-CPE	PS	Incorporation into the paste	DPV	12	10 d	Roasted coffee	FCA, DPPH	[101]
Lac/CBPE	TV	Cross-linking using GA and BSA	Amp	0.03	NR	Olive oil	FCA	[107]
Lac-PESM/DM/Pt	TV	NR	Amp	NR	NR	Wine	FCA, ABTS	[68]
Lac/SPCE	TV	MAPLE	Amp	1	100 d	Herbal extracts	FCA	[103]
Lac-PVA-AWP/SPCE	TV	Entrapment in PVA-AWP	Amp	0.524	−10%/2 m	Herbal tea	FCA	[60]
PPy/Lac/AuNPs/SPCE	NR	Entrapment in PPy	Amp	0.83	−15%/1 m	Propolis	FCA	[102]
Lac-nNafion/AuNPs/GNPLs-SPCE	RV	Entrapment in nNafion	Amp	1.5	5 d	Wine and syrup	ABTS	[71]
nNafion/Lac/rGO/PtNPs/SPCE	TV	Entrapment in nNafion	Amp	0.09	14 d	Herbal tea	FCA	[104]
Lac/GQDs/MoS_2_NFs/SPCE	TV	Adsorption	Amp	0.32	−15%/28 d	Wine	FCA	[105]
Lac-Nafion/SCE	TV	Cross-linking using GA, stabilization with Nafion	Amp	0.06	7 d	Beer	FCA	[66]
Lac/rGO-MWCNTs/GCE	TV	Cross-linking using GA annd BSA	Amp	0.3	15 d	Fruit juices	ABTS	[61]
Lac/rGO-GCS/GCE	TV	Cross-linking using GA	Amp	0.076	15 d	Herbal tea	FCA	[106]
Lac/GCE	TV	Cross-linking using GA	Amp, FIA-Amp	0.015, 0.235	15 d	Wine	FCA	[63]
Lac-PAP/MWCNTs/SPCE	TV	Entrapment in PAP	FIA-Amp	0.588	10 d	Wine	FCA	[64]
Lac-PAP/MWCNTs/SPCE	TH	Entrapment in PAP	FIA-Amp	1.763	10 d	Wine	FCA	[64]
Lac-PAP/SWCNTs/SPCE	TH	Entrapment in PAP	FIA-Amp	3.527	10 d	Wine	FCA	[64]
**Tyrosinase biosensors**								
Tyr/cPVC-G/cPVC-G-KMnO_4_	MA	Covalent binding with EDC	Potentiometry	0.73	3 m	Honey and propolis	FCA	[108]
Tyr/ClSubPc/ITO	M	Cross-linking using GA	CV	0.757	NR	Wine	NR	[70]
Tyr/PhOSubPc/ITO	M	Cross-linking using GA	CV	0.101	NR	Wine	NR	[70]
Tyr/*t*-BulSubPc/ITO	M	Cross-linking using GA	CV	0.21	NR	Wine	NR	[70]
Tyr/AuNPs/ESM/GCE	M	Cross-linking using GA	DPV	0.714	−7%/1 m	Tea and wine	HPLC	[109]
Tyr-CS/rGO-MWCNTs/GCE	M	Entrapment in CS	Amp	0.5	1 d	Fruit juices	ABTS	[61]
Tyr/AuNPs/GCE	M	Cross-linking using GA	Amp	0.15	18 d	Wine	FCA	[67]
Tyr-CS/MPNQ/Au	M	Entrapment in CS	Amp	0.019	−6%/7 d	Olive oil	NR	[110]
DM/Tyr-KCG/TM/Pt	NR	Entrapment in KCG	Amp	NR	NR	Wine	FCA	[112]
Tyr-PEDOT/SCE	M	Entrapment in PEDOT	Amp	4.33	10 d	Beer and wine	ABTS	[65]
Tyr/CBPE	AB	Cross-linking using GA and BSA	Amp	0.006	21 d	Olive oil	FCA	[107]
Tyr/AuNPs-SPCE	M	Cross-linking using GA	Amp	1.2	NR	Beer	FCA	[111]
Tyr/APTES/Fc-SPCE	M	Cross-linking using GA and BSA	FIA-Amp	4	30 d	Wine	NR	[113]
**Laccase–tyrosinase biosensors**								
Lac-Tyr-Nafion/SCE	TV, M	Cross-linking using GA, stabilization with Nafion	Amp	0.026	−6%/2 d, −17%/15 d	Beer	FCA	[69]
Lac-Tyr-DGS/APTES/Fc/SPCE	TV, M	Entrapment in DGS	FIA-Amp	2	−9%/2 d, −43%/5 d	Must and wine	FCA, I_280_	[116]

^1^ Lac, laccase; ClSubPc, hexa-chloro boron subphthalocyanine; ITO, indium tin oxide coated glass slide; PhOSubPc, hexa-phenoxy boron subphthalocyanine; *t*-BuSubPc, tri-*tert*-butyl boron subphthalocyanine; CuNPs, copper nanoparticles; CS, chitosan; cMWCNTs, carboxylated multi-walled carbon nanotubes; PANI, polyaniline; Au, gold; CA, cysteamine; AgNPs, silver nanoparticles; ZnONPs, zinc oxide nanoparticles; Fe_3_O_4_NPs, iron oxide(II,III) nanoparticles; NiNPs, nickel nanoparticles; CS-GAL, chitosan-galactomannan composite; 4ATP, 4-aminothiophenol; CPE, carbon paste electrode; CBPE, carbon black paste electrode; PESM, polyethersulfone membrane; DM, dialysis membrane; Pt, platinum; SPCE, screen-printed carbon electrode; PVA-AWP, polyvinyl alcohol azide-unit pendant water-soluble photopolymer; PPy, polypyrrole; AuNPs, gold nanoparticles; nNafion, ammonia-neutralized Nafion; GNPLs, graphene nanoplatelets; rGO, reduced graphene oxide; PtNPs, platinum nanoparticles; GQDs, graphene quantum dots; MoS_2_NFs, molybdenum disulphide nanoflakes; SCE, sonogel-carbon electrode; MWCNTs, multi-walled carbon nanotubes; GCE, glassy carbon electrode; rGO-GCS, reduced graphene oxide–glycol chitosan nanohybrid; PAP, polyazetidine prepolymer; SWCNTs, single-walled carbon nanotubes; Tyr, tyrosinase; cPVC, carboxylated polyvinyl chloride; G, graphite; KMnO_4_, potassium permanganate; ESM, egg shell membrane; MPNQ, ω-mercaptopropyl naphthoquinone; KCG, kappa-carrageenan; TM, Teflon membrane; PEDOT, poly(3,4-ethylenedioxythiophene); APTES, (3-aminopropyl)triethoxysilane; Fc, ferrocene; DGS, diglycerysilane. ^2^ TV, *Trametes versicolor*; GsR, *Ganoderma* sp. Rckk02; PS, *Pycnoporus sanguineus*; RV, *Rhus vernicifera*; TH, *Trametes hirsuta*; MA, *Musa acuminata*; M, mushroom; AB, *Agaricus bisporus*. ^3^ GA, glutaraldehyde; BSA, bovine serum albumin; MAPLE, matrix assisted pulsed laser evaporation; EDC, N-(3-dimethylaminopropyl)-N′-ethylcarbodiimide hydrochloride. ^4^ CV, cyclic voltammetry; DPV, differential pulse voltammetry; Amp, amperometry; FIA, flow injection analysis. ^5^ m, month(s); d, day(s). ^6^ FCA, Folin-Ciocalteu assay; DPPH, antioxidant capacity assay using 2,2-diphenyl-1-picrylhydrazyl; ABTS, antioxidant capacity assay using 2,2′-azino-bis(3-ethylbenzthiazoline-6-sulfonic acid); HPLC, high-performance liquid chromatography; I_280_, polyphenol index measured at 280 nm. NR, not reported.

## Data Availability

Not applicable.
